# The future of self-selecting and stable fermentations

**DOI:** 10.1007/s10295-020-02325-0

**Published:** 2020-11-02

**Authors:** Peter Rugbjerg, Lisbeth Olsson

**Affiliations:** 1 Enduro Genetics ApS, Copenhagen, Denmark; 2 Department of Biology and Biological Engineering, Industrial Biotechnology, Chalmers University of Technology, Gothenburg, Sweden

**Keywords:** Production load, Metabolic burden, Evolutionary stability, Production robustness, Production stability, Genetic heterogeneity, Phenotypic heterogeneity

## Abstract

Unfavorable cell heterogeneity is a frequent risk during bioprocess scale-up and characterized by rising frequencies of low-producing cells. Low-producing cells emerge by both non-genetic and genetic variation and will enrich due to their higher specific growth rate during the extended number of cell divisions of large-scale bioproduction. Here, we discuss recent strategies for synthetic stabilization of fermentation populations and argue for their application to make cell factory designs that better suit industrial needs. Genotype-directed strategies leverage DNA-sequencing data to inform strain design. Self-selecting phenotype-directed strategies couple high production with cell proliferation, either by redirected metabolic pathways or synthetic product biosensing to enrich for high-performing cell variants. Evaluating production stability early in new cell factory projects will guide heterogeneity-reducing design choices. As good initial metrics, we propose production half-life from standardized serial-passage stability screens and production load, quantified as production-associated percent-wise growth rate reduction. Incorporating more stable genetic designs will greatly increase scalability of future cell factories through sustaining a high-production phenotype and enabling stable long-term production.

## Introduction

To accelerate commercialization of the growing number of advanced bioproducts, new solutions are needed to minimize the development costs of scaling bioprocesses to large volumes [43, 63]. Often, large-scale biomanufacturing is restricted by the long-term stability of the production phenotype. Metabolic burdens and toxicities confer a production load (defined as percent-wise reduction in specific growth rate associated with production). As cultures are scaled to industrial numbers of cell generations (e.g*.* > 40 generations), this production load selects against high production and enriches for spontaneously emerging low-producing subpopulations [50, 66]. The economic consequences of a poorly scalable process can be significant with estimates of full bioprocess scale-up costs ranging between 100 million and 1 billion USD including costs of pilot and manufacturing plants [12]. In large bioproduction plants, physiochemical heterogeneity is another, well-described phenomenon that occurs as a consequence of suboptimal mixing gradients causing variation in substrate, oxygen and pH regulators [16, 57]. Cellular heterogeneity on the other hand emerges spontaneously and is enriched through selection. Two types of cellular heterogeneity can be delineated: genetic heterogeneity and phenotypic (non-genetic) heterogeneity. Genetic heterogeneity arises due to a variety of gene- and strain-specific mutation types [50], whilst non-genetic heterogeneity arises due to stochastic (noisy) gene and protein regulation, expression and distribution to daughter cells [3, 24]. Even when starting from a pure single-cell derived master clone bank, heterogeneities of low or non-producing cell variants can become significant in fermentations [8, 48].

Driving both non-genetic and genetic heterogeneity, the selective production load is very case-dependent and can be identified as a growth rate reduction that results from metabolic burden (e.g. molecular depletions in co-factors, redox, charged tRNA and ATP) [5, 26] and metabolic inhibitors such as intermediate and end product toxicity [62]. The collective load of production (production-associated fitness cost) can be quantified as the percent-wise reduction in specific growth rate due to production. A functional understanding of the causes of production load and possible mitigations can be obtained through omics approaches [35]. To quantify, understand and mitigate genetic heterogeneity, deep DNA-sequencing advances have lately enabled better insight into which mutational modes (rates, types and targets) that even simultaneously can dominate production heterogeneity [37, 48]. Non-genetic production heterogeneity has mainly been characterized using biosensors [41, 65], flow cytometry [16, 53], micro-engraving of individual cells [38] or microfluidics cultivations [28].

Recent synthetic biology strategies now promise to reduce both types of heterogeneity in long-term industrial cultures. This shift has enabled the development of synthetic product addictions and auxotrophies that couple growth to product formation. Product addictions can link growth to production via product or pathway-sensitive biosensors that regulate growth by either constitutive essential genes or conditional selection genes [14, 39, 49, 61, 65] with different benefits. Synthetic product auxotrophies can be generated by metabolically linking pathway flux to essential growth-coupled parts of the metabolism [30].

Better strategies are also needed for deconvoluted monitoring of long-term robustness and susceptibility to heterogeneity. For example, the productive lifetime of populations increases when the initial production load is lowered [48]. However, the inherent positive correlation between production loads (burden and product toxicity) and production titers, rates, yields (TRY) is masking the picture [32, 38]. As a consequence, improvements in production load may result from drops in TRY, and initially higher-performing strain variants can turn out to perform worse after several cell generations compared to less loaded lower-producers [56]. Therefore, to improve the design of scalable strains it is important to monitor and address production load as well as the formation rate of spontaneous heterogeneity. As a result, predictors of long-term robustness, the production load and production half-life may be of similar importance to the TRY metrics.

To better predict the process scalability of new pathway designs in academia and industry, we suggest: (1) routine, comparable stability screens to measure production half-life, and (2) routine measurements of production-associated decrease in specific growth rate (i.e. fitness cost) to infer production loads and predict stability early in the design-build-test cycles of future cell factories. We further discuss synthetic biology stabilization strategies and classify them as directed towards the mutation genotype or the production phenotype.

## Predicting and quantifying production half-life

Routine monitoring scale-up stability of new strains using standard methodology will likely enable strain engineers to better prioritize scalable early designs for example compared with short-term improvements that lack long-term robustness. Strain stability can be predicted by the direct production load through relative specific growth rate measurements of producing variants and low/non-producing variants, or the production half-life can be measured in serial-passage stability screens [48, 56]. Here we outline these two metrics and provide principles for experimental design to more accurately grasp the physiological conditions of eventual industrial production including seed train propagation.

### Production half-life estimation by serial passaging

**Fig. 1:**
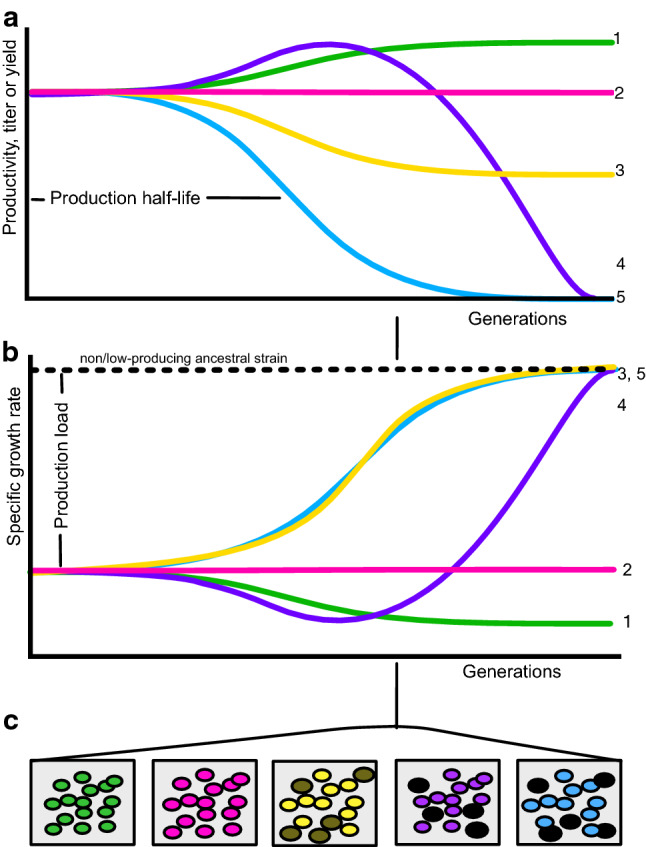
Production over time may change due to non-genetic and genetic heterogeneity as well as homogenetic change. Five hypothetical heterogeneity scenarios are presented in relation to, **a** bulk-population performance, **b** bulk population-specific growth rate and c) population composition with regards to producing cells (colored), low-producing (gray-colored) and non-producing cells (black-colored). Over a typical cultivation time line, these scenarios may be characterized by: case (1) stable increase in performance and increase in load (e.g. by homogenous, population-uniform change) (green-colored), case (2) stable performance despite a production load (e.g. by a low spontaneous formation rate of heterogeneity) (pink-colored), case (3) partial decline to intermediate lower level due to rising subpopulation that overcame the production load through producing at lower level (e.g. [55]) (yellow-colored), case (4) homogenous increase in performance that increases the production load, in turn leading to higher enrichment rate of heterogeneity (purple-colored), case (5) enrichment of heterogeneity driven by a load, leading to complete production decline (e.g., [27]) (blue-colored)

Serial passaging of cultures can simulate long-term industrial cultivations to measure production half-life as the number of generations at which half of the initial production level is reached (Fig. 1a). In industrial production, seed trains are used to inoculate large fermenters and manage cell generations in a production process. In industry seed trains are usually passaged prior to reaching stationary phase to avoid subsequent lag phases [29]. While intermittent stationary phases have direct impact on subsequent growth behavior, the stationary phase can in some cases also trigger different mutational responses [22]. Importantly, industrial practice can also entail different stages such as using inducers and feeding programs which may largely change the production load depending on the type of product. While cells are already stressed during production, stationary phases instigate an unwanted stress response and should be avoided when simulating scale-up. Further, the passage (seed) volume not only influences the practical timing, but potentially also the simulation of long-term cultivation if population bottlenecks are introduced [7] e.g. by passaging markedly fewer cells than the expected mutant formation rate. Therefore, production half-lives can be determined in serial-passage experiments that resemble adaptive evolution experiments (ALEs) in methodology [52]. However, care should be taken to design passaging regimes that avoid unrealistic stationary phases, to allow comparison between academic studies and industrial practice [29]. Since the estimated production half-life ultimately depends on the formation rate of spontaneous heterogeneity, the size of the production load and the quality of the scale-up mimic, it can be difficult to compare an individual factor between different studies. Nonetheless, serially passaging of 0.2–2% volume from growing (non-stationary phase) cultures appear suitable as standard principle. However, lab-scale serial passaging does often not simulate the significant shear pressure and oxygen or nutrient heterogeneities of large-scale bioreactors. A relevant solution could be to perform serial-passage simulations in scale-down systems.

### Production half-life estimation by measuring production load

Measuring the relative fitness cost of production by specific growth rates (production load) is a simple way to predict the potential stability of constructed strains. The specific growth rate of the strain should be measured in relevant production medium and compared to the ancestral non-overproducing strain, for example containing an empty vector, or no integrated heterologous genes (Fig. 1b). Specific growth rates can be measured in a microtiter plate system, but any changed aeration, pH, temperature and similar conditions relative to the end process will potentially bias the results. The specific growth rate reduction may be specific to particular stages in the production cultivation (for example if a particular substrate is fed late), and this will complicate the ability to predict stability. A very interesting alternative may be monitors of cellular capacity (or “residual expression capacity”) measured as change in (otherwise) constitutive green fluorescent protein (*gfp)* expression to quantitate expression burden with single-cell compatibility [9]. However, only measuring expression burden may not capture relevant contributions to the production load from metabolic toxicities and inhibitions.

## Non-genetic and genetic heterogeneities affect bioprocess performance at scale

Different simultaneous types of heterogeneity can be expected in long-term fermentations. Further, in addition to heterogeneity-driven changes, homogenous changes may also occur in cultures over time, e.g. through non-genetic inheritance of the proteome, thereby further affecting the population dynamics. Therefore, to estimate the productive lifetime of a process, it may be helpful to consider theoretical scenarios of production dynamics. The S-shaped, bimodal loss curve has been reported in numerous examples [50], and is easily modeled mathematically [47] (Fig. 1—case 5). However, heterogeneity in cultures can also reduce production levels only partially depending on the available routes for alleviating production load (Fig. 1ab—case 3), e.g. as seen in early chemostat-based studies [2, 55]. In addition to changes driven by cell heterogeneities (e.g. emerging non-producer cells), temporal changes in production performance may potentially also be strictly homogenous (i.e. uniform across the bulk population). Observed at bulk-population level, changes in protein-composition during long-term cultivation e.g. affected insulin precursor production in *S. cerevisiae* expressed from a multi-copy plasmid [34, 64]. In the case of ethanol-producing *S. cerevisiae*, short-term adaptation to the medium during propagation improves the quality of the propagated cells leading to an improved overall ethanol productivity [18], suggesting a positive role of adaptation to the environment when producing a primary metabolite, e.g. by increasing cell viability. Non-genetic heterogeneity can revert as indicated in l-valine producing populations of *C. glutamicum,* where L-valine concentrations were visualized with a product biosensor coupled to fluorescent protein [41]. Non-genetic and genetic heterogeneity in production cultures has largely been studied using different techniques and profiled in different studies and production systems [41, 48]. However, no evidence suggests a mutual exclusivity, and both types of cell heterogeneity are likely affected by similar selective forces. In addition to the effect of population dynamics on bioprocesses, a number of studies have reported temporal production declines characterized by changes in protein composition [34]. It is important to keep in mind that these changes may be homogenous and thus not necessarily driven by more-fit subpopulations. However, it is possible that epigenetically inherited protein will homogeneously decrease the specific growth rate and thereby increase the production load (Fig. 1—case 1). In such cases, increasing production load may exacerbate the subsequent enrichment of cell heterogeneity (Fig. 1—case 4) and also complicate the ability to predict stability based on the initial production load. To separate these types, in general, it can be expected that heterogeneity-driven performance changes will be indicated by increases in specific growth rate due to selection, while decreases in specific growth rate are expected for homogenous changes in performance. However, heterogeneity in cultures can also partially reduce production levels, depending on the available routes for alleviating production load (Fig. 1ab—case 3), e.g. as seen in chemostat-based assessments [2, 55].

## Mutation genotype-directed strategies prevent production declines leveraging DNA-sequencing

**Fig. 2:**
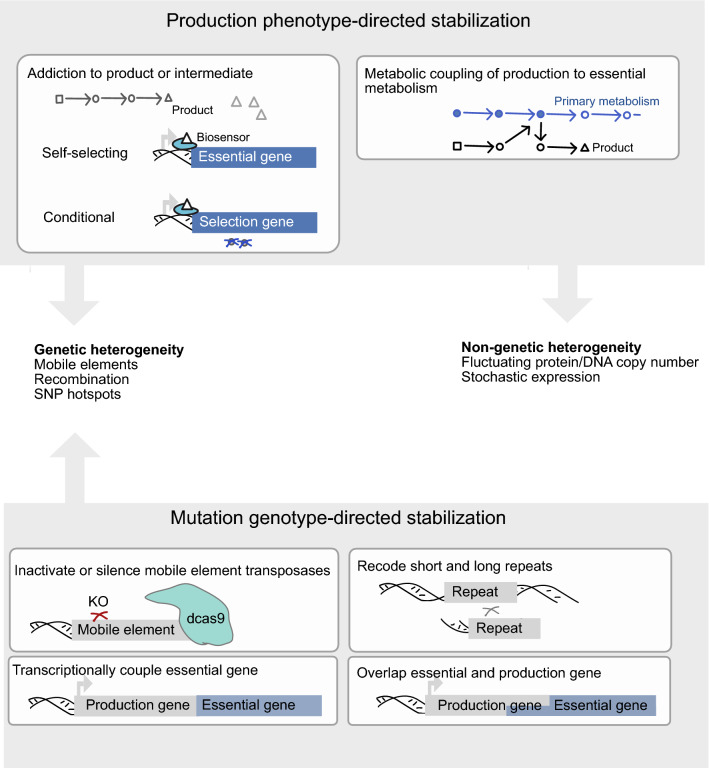
Strain engineering strategies for stabilization of long-term bioprocesses. Stabilization of long-term bioprocesses can be divided into mutation genotype-directed (bottom-up) strategies mitigating known recurring mutational genotypes e.g. informed by DNA-sequencing, and production phenotype-directed (top-down) strategies that provide a non-natural growth advantage to a desirable production phenotype, e.g. by coupling the pathway/product metabolically to essential metabolism or sensing for the pathway/product using genetically encoded biosensors

Faster and cheaper deep DNA-sequencing together with improved bioinformatics tools now make it feasible to profile mixed genetic subpopulations. This can for example visualize different parallel escape paths in production genes or uncover if one particular mutation event is highly active. To economize sequencing reads, an initial analysis can be directed towards the engineered genes. It can be difficult to generate a mutational profile at significant population depth, especially below 1% population frequencies where many sequencing error types become significant [50]. Early studies of cultivated production populations indicate that certain mutational modalities appear more often than others [48, 56]. Such case-specific knowledge from DNA-sequencing can inform mutation genotype-directed stabilization strategies (Fig. 2) (Table 1). In prokaryotic workhorses *E. coli, Corynebacterium glutamicum* and *Bacillus subtilis*, particular subfamilies of insertion sequence (IS) elements e.g. appear to decimate load-carrying genes for production more quickly. IS elements are bacterial mobile genetic elements that transpose autonomously in a host genome through the action of self-encoded transposases. This discovery has led to the development of platform strains with reduced mutation rates through lowered IS activity (Table 2), but other escape paths appear frequently, with recombination being particularly noticeable and even facilitated by multiple copies of IS elements in different chromosomal locations [50].

**Table 1:** Detected pathway mutations and mutation genotype-directed stabilization strategies

Mutation type	Host and product	Suggested stabilization strategy	Reference
SNPs, deletions	*E. coli* threonine deaminase	Essential gene fusion (sequence entanglement)	[4]
IS	*E. coli* mevalonate	Essential gene fusion (transcriptional coupling)	[48]
Large duplication	*E. coli* 1,4-butanediol	Deletion of recombinogenic IS repeats	[8]
Deletions	*E. coli* fluorescent protein	Avoid DNA repeats	[56]
IS	*A. baylyi* fluorescent protein	CRISPRi knockdown of IS	[25]
IS	*C. glutamicum* fluorescent protein	IS deletion	[10]
Recombination	*E. coli ﻿*poly-3*-*hydroxybutyrate	*recA* deletion	[58]

**Table 2:** Optimized host organisms engineered to reduce effects from mutation-causing genes

Organism	Targeted mutagenic component	Strategy	Reference
*E. coli* MDS42	ISs, prophages	Deletion of all ISs and prophages	[46]
*E.* coli	ISs	CRISPR-based abolishment by point mutation, conjugation of IS-less regions	[59]
*E. coli* TOP10	ISs	CRISPRi knockdown of IS	[25]
*E. coli* MG1655, BL21(DE3), DH5-alpha, JM107MA2	ISs	CRISPRi knockdown of IS	[44]
*Bacillus subtillis*	ISs	Deletion	[40]
*Corynebacterium glutamicum*	ISs, error-prone DNA polymerase	Deletion	[10]
*Acinetobacter baylyi*	IS	CRISPRi knockdown of IS	[25]

### Removal or knockdown of transposable elements such as ISs

In bacterial production organisms, mobile genetic IS elements are a known nuisance to production. IS elements can transpose into engineered production genes or facilitate recombination [8, 50]. These elements can provide an evolutionary advantage in changing environments by offering reversible knockout of random genes [21], but a similar mechanism is not advantageous in a bioprocess perspective. Avoiding disruption due to IS elements is a frequent priority, also due to examples of wide dissemination in genomes of popular key host strains such as *Escherichia coli* DH10B (66 copies) [19]. It can be cumbersome to systematically delete all IS elements and new transposition can occur in the process [46] (Table 2). Improved mitigation strategies employ CRISPR-based abolishment and genetic conjugation to make hybrid genomes from IS-less genomic regions in related strains [59]. Lately, also strategies using CRISPRi to silence the transposase genes driving the IS have been shown for *E. coli* strains and *Acinetobacter baylyi* [25, 44]. Silencing strategies are fast and easy to test, yet actual deployment of CRISPRi during production is likely a major burden in itself [13] with risk for quick mutation and indirect metabolic costs to biosynthetic production. To further develop such an approach, IS silencing could be established for metabolite production and at longer time scales than fluorescent protein demonstrations so far shown. On the positive side, far from all chromosomal IS subtypes appear highly active. Promoted by different stresses, temperatures and other signals, it has been shown that only a subset of the wide IS subtypes are typically active [36], which could likely reduce the number of IS removals needed. The genome-reduced, IS-free *E. coli* MDS42 strain has also been shown to improve stability, though it has been suggested that the vast genome reduction is not necessarily beneficial to the industrial exploitation of its metabolism [20, 48].

### Removal of insertion sequence target sites

Recoding the usual 6–9 base pairs long target sites of the IS elements poses an alternative IS mitigation strategy that has been rarely exploited to date. This is likely due to the numbers involved and the fact that the target site specificity is usually sufficiently broad to not drastically prevent transposase entry in the engineered gene sequence.

### Direct coupling or overlapping of essential and product formation genes

Several stabilization strategies physically couple or overlap essential genes with the product formation gene to maintain it (Fig. 2). This is similar to transcriptional or translational fusion of selectable genes such as tetrahydrofolate reductase genes used in the improvement of protein production [1]. Yet, the necessary selectable agents are not feedable during production e.g. due to cost and/or toxicity. Protein production benefits from physical coupling by working directly on the target gene for the protein in question, whereas stabilization of metabolite production is complicated by the need for balancing multiple enzymatic activities [31]. Alternative non-conditional systems have been shown even in mammalian systems using an *E.* coli antitoxin co-expressed with the protein product to overcome simultaneous toxin expression [42]. To stabilize burdensome expression of a metabolic pathway, Rugbjerg et al. [48] previously transcriptionally coupled the *E. coli* essential gene *murI,* transferring it from the chromosome to the plasmid-localized mevalonic acid pathway operon. The hypothesis was that IS insertions would shut down transcription on the downstream essential gene, whose presence would thereby maintain the pathway intact [48]. Yet, the long-term production stability improved only modestly since competing IS elements circumvented the solution. This was likely allowed through exhibiting a less growth-restrictive influence on the coupled essential gene.

Appearing more broadly effective is the sophisticated engineering of direct sequence overlaps, “gene entanglements “between a foreign (production) gene and an essential gene [4]. The strategy exploits the natural concept of prokaryotic overlapping genes and aims to artificially overlap the two gene sequences by shared sequence enabled by the redundant codon space. Blazejewski et al. [4] in silico predicted the sequence space of possible sequence overlaps between an essential and foreign gene. Given the wide sequence space of potential entanglements, applying such strategies in metabolite or protein production using entangled production genes may be possible. However, it may be challenging to balance the ideal expression strength for a balanced metabolic pathway gene with the expression strength of an entangled essential gene without compromising fitness in adverse ways to the process.

### Are plasmid-encoded genes more susceptible to mutation?

Plasmid-based expression is still often used industrially, e.g. via the pET series [54], due to the ease of attaining many gene copies. The loss of plasmid without selection (segregational instability) has been solved by antibiotics-free plasmid addiction systems in industry [23] or through kanamycin selection, which can sometimes be accepted by the authorities regulating the process. However, structural instability (susceptibility to structural variation) and other types of mutational instability will still affect heterologous or engineered genes and the rates may depend on way they are propagated in the cell [58]. The random partitioning of plasmids during cell division may amplify single mutation events favoring the growth of cells that contain a high proportion of mutated variants in presence of selection. This process of mutant enrichment via segregational purification has mainly been studied in systems absent of plasmid selection, rendering multi-copy chromosomal integrations more stable [58]. Yet, multi-copy chromosomal integration also appears to prevent clonal amplification of escaping alleles due to uneven, random plasmid partitioning [58]. Effectively, uneven segregation, in the face of a plasmid-borne fitness cost, means that plasmids de facto pass on novel variants as near single copy, yet has a mutational supply of corresponding to the plasmid copy number [51]. However, it remains to be shown how wide this effect is, including its dependency on different plasmid origins and selection pressure. As exemplified in *S. cerevisiae* based production of vanillin-β-glucoside, chromosomally integrated genes are also the target of rapid mutant enrichment if provided with a production load [14].

In *E.* coli producing GFP, a clever strategy to reduce general mutation frequencies was outlined using flow cytometry and sorting for mutant cells with maintained high GFP expression following serial cultivation [15]. The authors expressed GFP from a high-copy ColE1-type plasmid and this strategy identified a *polA* polymerase mutation which decreased plasmid mutation frequencies. As side effects, GFP levels and plasmid copy numbers decreased, making it challenging to isolate the effects of a reduced mutation rate.

Therefore, successfully lowered mutation rates can be difficult to isolate from any co-occurring, but secondary decrease in expression strength that could be counteracted afterwards. The authors therefore used a model to isolate significantly lower mutation rate from the reduced fitness cost also resulting from the lowered copy number. Interestingly, the authors [15] did not report structural variant error modes (such as IS element or recombination) as escape mode on this plasmid. Similarly using the power of flow cytometry, the same group also investigated incQ-type plasmids and isolated the emergence of parasitic satellite plasmids that shed the replication genes and expression constructs [67]. Even if uneven segregation of multi-copy plasmids elevate escape rates, single chromosomal gene copies can also be highly vulnerable, as shown through sequencing of vanillin-beta-glucoside producing *S. cerevisiae* [14].

## Self-selecting and phenotype-directed strategies that couple production to growth

Using recent synthetic biology, high-performing fermentation populations can be long-term stabilized by strategies that couple a desirable production phenotype to the specific growth rate at a single-cell level. Leading towards a concept of self-selecting fermentations, these strategies therefore do not require knowledge on the specific escape paths as the strategy is directed towards presence of a particular product or important intermediate on its metabolic pathway. Production phenotype-directed stabilization can result through coupling production directly to essential metabolism [33, 60], or through product addictions employing genetically encoded product-sensing biosensors (Fig. 2). Biosensors then detect the product or intermediate and next direct this signal to activate expression of genes essential for growth. For some of the growth-controlling genes used in studies, essentiality depends on the conditions; product addictions have been designed using classical antibiotics or auxotrophy-based selection genes [14, 39, 65], and using self-selecting, non-conditionally essential genes [49] (Table 3).

**Table 3:** Published production phenotype-directed synthetic addictions, including systems relying on conditioned medium

Organism	Product	Production stability (> 90%) (cell generations)	Growth controlling gene	Selective condition	Reference
*E. coli*	Fatty acids Tyrosine	No long-term serial-passaging cultivation	Leucine prototrophy *leuABCD* Antibiotic resistance gene *tetR*	Leucine depleted, Tetracycline	[65]
*E. coli*	Mevalonic acid	95	Folate and peptidoglycan biosynthesis *folP*-*glmM*	None (self-selecting)	[49]
*E. coli*	Tryptophan Phenylalanine	No long-term serial-passaging cultivation	Toxin-Antitoxin system *hipA*-*hipB*	None (self-selecting)	[61]
*S. cerevisiae*	Vanillin-ß-glucoside	55	Glutamine prototrophy *GLN1*	Glutamine depleted	[14]
*Y. lipolytica*	Naringenin	320	Leucine prototrophy *LEU2*	Leucine depleted	[39]

The reported stability of the production pathway is reported but the instability of the production phenotype also depends on the product (not comparable due to lacking production load measurements)

### Synthetic product addictions by pathway biosensors coupled to selection genes

In an early study to enrich non-genetic higher-producing cell variants, product-sensing biosensors were coupled to selection genes in *E. coli* cells, resulting in better fatty acid and tyrosine production during the cultivation time scale of a bench-top fed-batch fermentation [65] (Table 3). Xiao et al. [65] thereby underlined a significant optimization opportunity of non-genetic heterogeneity even within very short cultivation (likely around 30 cell generations and production only induced at the end). The study was therefore not investigating long-term bioproduction in terms of further cell generations, which can be done by serial passaging. The authors applied classical selection genes for leucine prototrophy and tetracycline resistance to control specific growth rates, as these genes become essential in medium conditioned to these selections. This concept (“PopQC”) led to a remarkable fourfold increase of free fatty acid titer to 22 g/L [65]. Such short induction and cultivation time scales clearly appear fit for enriching non-genetic variation while genetic variants generally require longer time scales [11, 27, 49]. An advantage when controlling populations using classical conditional selection genes is that designs can be more easily engineered, simply through using the selective medium conditions [14, 39, 65]. Conditional selections also enable tuning of the selective pressure by changes in concentration of the selective agent, similar to biosensor-based enzyme screening [17]. Finally, selection genes allow time-dependent control of populations for example if initial intracellular fatty acid product concentrations were too low to activate the biosensor and maintain growth [65]. Such timing was also needed to control populations of *S. cerevisiae* producing vanillin-ß-glucoside, in which metabolic addiction was engineered using the glutamine biosynthetic gene *GLN1* as selection gene. Glutamine biosynthesis was coupled to biosensors sensing pathway intermediates, and initial cultivation was carried out without selection in rich medium, as initial cultures would not grow without the growth-controlling glutamine [14]. A few challenges may speak against the use of classical selection genes, as medium conditions such as antibiotics and dropouts can be industrially challenging to introduce. Further, as discussed by Lv et al. [39], use of classical selection genes (i.e. involving nutrient auxotrophy and antibiotics resistance) also comes with the risk of cross leaking the controlled nutrient (e.g., glutamine or leucine) or enzyme (e.g. beta-lactamase) to cheating cells [39]. Lv et al. instead built a naringenin-addicted production host in *Yarrowia lipolytica* using leucine biosynthetic gene and extended the production stability of naringenin production from approx. 200–320 generations. Though not observed so far, the cross-leakage scenario or “microbial herd protection” could mean that low-performing subpopulations are permitted by feeding from cross-leaking cells until they reach a certain fraction of the population. Similarly, cross leakage of product to lower-producing cells has been highlighted as a risk for circumvention of the sensing genes. This scenario is particularly likely towards the end of fed-batch fermentations in which the intracellular concentrations are likely elevated. This would present cells with an opportunity for escaping production, yet the space for taking over the population would be limited by reduced growth towards the end of the fermentation. To mitigate this problem, sensing for a critical toxic pathway intermediate may be a beneficial strategy such as shown when stabilizing the pathway to vanillin-ß-glucoside [14].

### Self-selecting fermentations by synthetic product addictions in selection-free medium

Instead of engineering addictions using a potentially cross-leaking metabolite or toxin, non-conditional (self-selecting) addictions control growth rates using constitutively essential genes (Fig. 2) [49, 61]. In self-selecting addiction systems (i.e. systems independent on media depletions/supplements), essential genes encoding for example cell wall biosynthesis and antitoxins are utilized (Table 3). Such addiction systems thereby connect the sensed phenotype with a constitutive selection pressure. Identification of suitable constitutively essential genes can be challenging. These should not result in pleiotropic effects on the metabolic production pathway, yet production cross talk can relatively easily be screened for. Untoggled selection is not possible with constitutively essential genes, and this complicates the construction of constitutively product addicted strains. However, self-selecting product addicted strains promise a longer stability and process adaptability by circumventing media conditioning and cross leakage. Both types of product addiction systems represent an added metabolic and transcriptional load that could negatively influence baseline performance of the controlled cell factory strain. Therefore, it will be important to assess and avoid any such load resulting from the system during the development phase.

### Synthetic auxotrophy by direct metabolic coupling of production to growth

Metabolic coupling between a loaded heterologous pathway and the essential metabolism is another interesting stabilization strategy directed to the production phenotype. The resulting auxotrophy for the product or intermediate generates a phenotypic pull, which has been used to select for better producing strains, for example through ALE [11]. The principle can be applied through knockout of particular metabolic reactions, forcing flux through the heterologous pathway. Genome-scale metabolic modeling in five main production organisms has shown that growth coupling is possible for a wide variety of pathways, at least upon several gene deletions [33], but growth-coupling strategies can also involve gene insertions and medium supplements such as antimetabolites that inhibit core reactions for example as used in selections during strain construction [6, 30]. As example, metabolic coupling to growth has been designed for anthranilate production from glycerol in *E. coli* by deleting four genes in the pyruvate-releasing enzymes of the central metabolism, thereby forcing the strain to synthesize anthranilate [60]. Similarly, synthetic mevalonate auxotrophs have been designed in *E. coli.* These were used as whole cell reporters in screens for improved mevalonate synthesis rather than to optimize production or stability [45]. Overall, metabolic coupling appears promising but to our knowledge effects on stabilizing phenotypes over time still remains to be demonstrated in the literature. One first relevant challenge in this regard may be improving the specific growth rates of the growth-coupled mutants to ensure better stability and avoiding unfeasible medium supplement conditions and easily disrupted gene insertions.

## Outlook towards enabling genetically stable continuous bioprocesses

Driven by the recent advances in synthetic biology, metabolic engineering and next-generation sequencing, many more innovations are likely to increase long-term bioprocess stability as we learn more about the possible production escape paths. An important first step will be more routine measurements of the percent-wise growth reduction due to production (production load) as well as production half-life measurements through serial-passaging experiments. Particularly transformational strategies will reduce the burden and inhibitions underlying the production load and use synthetic biology tools for regulating cellular growth and production. Both strategies directed to the mutation genotype and production phenotype are likely to contribute towards making engineered production organisms more easily scalable to large cultivation volumes. While not yet shown, self-selecting fermentations will in principle also allow enriching for beneficial genetic variation. Due to the comparably lower rates of spontaneous mutation than phenotypic variation, enrichment of beneficial genetic variants would likely require longer than the typical 100 generations of cultivation to improve bulk-population level production. This would therefore rarely happen during industrial fed-batch production, but the principle could be used in development of more stable genetic variants at lab scale. As the fields of metabolic engineering and bioproduction are developing with the onset of many new sophisticated bioproducts, it is becoming increasingly realistic to design strains early on that will exhibit long-term stability. Future studies will hopefully allow for more routine evaluation of the scalability of new promising strain designs, and e.g. also answer questions related to how widespread heterogeneity-driven production decline is in contrast to homogeneous long-term adaptation in production organisms. Excitingly, the recent developments will make continuous production an interesting alternative to the current, industrially dominating fermentation modes.

## Glossary

Biosensor: A genetically coded transcription factor or RNA switch that senses a metabolite through binding and in turn regulates gene expression.
Gene fusion strategies: Synthetic stabilization strategy for a product formation gene either by overlapping or non-overlapping the gene with an essential gene. Overlapping strategies are engineered by (partially) overlapping its nucleotide sequence with the sequence of an essential gene in another reading frame thus exploiting the redundancy of the genetic code [4]. Non-overlapping strategies transcriptionally or translationally couple the product formation gene and essential gene [1, 48].
Metabolic burden: Generally in metabolic engineering, it describes metabolic depletions and cross-talk associated with production and e.g. resulting in reduced specific growth rate [26].
Micro-engraving: A single-cell cultivation technique employing microwells, each approx. 0.05 mm in diameter [38].
Plasmid addiction: A synthetic stabilization strategy for a plasmid, preventing segregational instability (plasmid loss) but not structural instability. The strategy renders presence of a helper gene on the plasmid essential for growth [23].
Population bottleneck: A reduction of the population size to a very low number of members, which may bias the simulation of a long-term fermentation through lowering of genetic diversity [11].
Product addiction: A synthetic stabilization strategy for a metabolic product, in which a product-sensing biosensor senses high product concentration and in turn activates an essential gene [49] or a conditional selection gene [65].
Production load: The relative reduction in specific growth rate associated with production due to all factors selectively impacting producing cells in a given process.
